# Experimental Study on the Performance of Graded Glass Fiber Reinforced Concrete (G-GRC) Based on Engineering Application

**DOI:** 10.3390/ma14051149

**Published:** 2021-02-28

**Authors:** Qingbiao Wang, Hongxu Song, Yue Li, Fuqiang Wang, Zhongjing Hu, Shumei Lou, Zhenyue Shi

**Affiliations:** 1 College of Energy and Mining Engineering, Shandong University of Science and Technology, Qingdao 266590, China; skd990748@sdust.edu.cn (Q.W.); 201983010016@sdust.edu.cn (H.S.); 2 College of Safety and Environmental Engineering (College of Safety and Emergency Management), Shandong University of Science and Technology, Qingdao 266590, China; huyang@sdust.edu.cn; 3 State Key Laboratory of Mining Disaster Prevention and Control Co-Founded by Shandong Province and the Ministry of Science and Technology, Shandong University of Science and Technology, Qingdao 266590, China; 4 National Engineering Laboratory for Coalmine Backfilling Mining, Shandong University of Science and Technology, Tai’an 271019, China; 5 College of Resources, Shandong University of Science and Technology, Tai’an 271019, China; 6 College of Civil Engineering and Architecture, Shandong University of Science and Technology, Qingdao 266590, China; 201983040032@sdust.edu.cn (Y.L.); 201983040061@sdust.edu.cn (F.W.); 7 Department of Intellignt Equipment, Shandong University of Science and Technology, Tai’an 271019, China; skd992951@sdust.edu.cn

**Keywords:** alkali-resistant glass fibers, fiber reinforced concrete, compressive strength, tensile strength, flexural strength, impervious performance

## Abstract

An important way to improve concrete performance is the use of alkali-resistant glass fibers (ARGFs) as reinforcement. This paper is based on the problems of the cracking of the partition wall and lining seepage in Laoshan Tunnel, Qingdao, China. Two types of ARGFs were selected as reinforcement materials for the partition wall and lining concrete: high dispersion (HD) and high performance (HP); and the compressive strength (CS), tensile strength (TS), flexural strength (FS), and impervious performance (IP) of concrete with different gradations of the two types of fibers were investigated. The results show that although the CS of graded glass fiber reinforced concrete (G-GRC) is slightly decreased, the TS, FS, and IP of G-GRC are significantly improved. When the densities of the ARGFs of HD and HP are 0.6 and 5 kg/m^3^, respectively, G-GRC performs best; additionally, compared with ordinary concrete, the TS, FS, and IP of G-GRC are increased by 15.86%, 14.90%, and 31.58%, respectively. Meanwhile, the tension–compression ratio is increased by 22.29%, and the mechanical properties of concrete are remarkably enhanced. The research results were successfully applied to the construction of the Laoshan tunnel, and good engineering results were obtained.

## 1. Introduction

**Figure 1:**
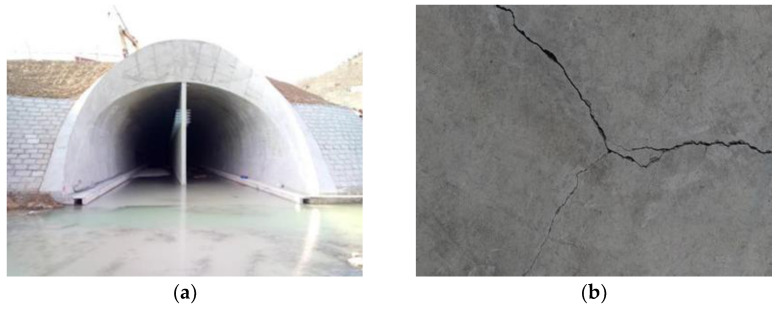
Scenic pictures of Qingdao Laoshan Tunnel: (**a**) Features of the middle partition wall; (**b**) cracking of the middle partition wall.

Good compressive strength (CS), tensile strength (TS), flexural strength (FS), and impervious performance (IP) are the inevitable requirements of engineering construction, and fiber reinforced concrete is one of the ways to improve these various factors of concrete under the premise of not improving the cement mark, which can reduce the construction cost to a certain extent. The idea for this article originated from the construction of the partition wall of the Laoshan Tunnel in Qingdao, China. As shown in Figure 1, the partition wall is long, large, and thin; hence, cracks can easily form in the partition wall during the construction process. To solve this problem and control costs as much as possible, the team provided a fiber reinforced concrete solution; meanwhile, the application was extended to part of the tunnel lining construction.

Presently, commonly used concrete-reinforcement fibers include steel fiber and polypropylene fiber, among other materials; however, these have problems such as weak durability, precipitation, or clustering after mixing. The density of alkali-resistant glass fiber (ARGF) is equivalent to that of concrete, with excellent dispersibility in concrete, and ARGFs are a new type of green and environmentally friendly reinforcing material. On the physical level, owing to the high strength and strong bondability with concrete of ARGFs, the TS and FS of concrete can be greatly improved. At the chemical level, owing to the addition of alkali corrosion resistant zirconia in the auxiliary material, a layer of corrosion-resistant protective film forms on the surface of the fiber, which greatly improves its durability in concrete. The results of the China Research Institute of Building Materials show that after 360 days of an accelerated aging experiment, the retention rate of ARGF was still over 88.5%, and the fiber surface remained smooth, as observed via SEM. By analyzing the corrosion trend of fibers, it is inferred that the safe service life of ARGF cement products can exceed 100 years [1]. The literature [2,3,4] also studied and verified the corrosion resistance mechanism of the fiber, the long-term strengthening effect on concrete, and durability. Based on its good physical properties and stable chemical properties, ARGFs have recently become the most widely used and fastest developing concrete reinforcement material. Therefore, many studies have been conducted on the content, mechanical properties, and mechanism of action of ARGF reinforced concrete.

ARGF reinforced concrete research aspects include the following: Yang et al. [5] conducted experimental research on reinforced coral aggregate concrete with different ARGF contents regarding the compression, tensile, and flexural resistance and verified the good physical strengthening performance of ARGF. Tassew et al. [6] studied the mechanical properties of glass fiber reinforced ceramic concrete through laboratory tests, and the results showed that it had good workability and mechanical properties. Shi et al. [7,8,9,10,11] through mechanical experimental research, revealed the effect of ARGF as an admixing material on the performance of concrete grouting materials, and it has been used in coal mines. Nourredine [12] studied the effects of curing conditions on the durability of cement-based ARGFs using SEM and XRD techniques. Moceikis et al. [13] studied the effect of aggregates on the mechanical properties of ARGF reinforced concrete. Wang et al. [14] studied the influence of high temperature on the strength and thermal conductivity of glass fiber reinforced concrete (GRC), revealed the performance variation rule of GRC under high temperature, and laid a theoretical foundation for the development and application of high-temperature-resistant GRC. Hybrid fiber reinforced concrete research aspects include the following: Liu et al. [15] studied the strengthening effect of ARGFs and polypropylene fibers on the mechanical properties of concrete through compressive and flexural tests and evaluated the durability of fiber reinforced concrete through a rapid chloride migration test and rapid chloride penetration test. Prathipati et al. [16] explained the uniaxial behavior of hybrid graded fiber reinforced concrete with glass and steel fibers and proved through experiments that the strength, toughness index, and ductility of mixed reinforced concrete are significantly improved. Wang et al. [17] studied the reinforcement effect of glass fiber and carbon fiber on the mechanical properties of fiber reinforced polymer concrete through experiments. Wu et al. [18] experimented and studied the effects of polypropylene fiber and glass fiber with different volume fractions on the mechanical properties of peach shell lightweight concrete and proved the enhancement effect of two kinds of fiber mixing on the mechanical properties of the concrete. Noh et al. [19] conducted an experimental study on the engineering properties of carbon fiber and glass fiber reinforced recycled polymer concrete. The advantages of these concretes in hydraulic structures and underground facilities were verified. Algburi et al. [20] prepared fiber reinforced reactive powder concrete using steel and glass fibers and studied the mechanical properties of fiber reinforced reactive powder concrete through experiments.

The literature shows that although many scholars have studied ARGF reinforced concrete and gained rich research achievements, they focused on a single type of ARGF (such as single high-dispersion (HD) fiber or high-performance (HP) fiber) and multiple types of hybrid fibers (such as glass fiber and polypropylene fiber) and their enhancement effect on concrete. There are few studies on the strengthening effect of a mixture of different types of ARGFs (the same fiberglass but different models, such as HD and HP). Different types of ARGF mixtures are complementary in strengthening concrete, but their effects on the mechanical properties of concrete remain unclear. Therefore, it has important practical value to study the influence of different types of ARGF mixtures on the mechanical properties of concrete.

The different types of ARGFs, mixed into concrete in a certain ratio, are called graded GRC (G-GRC). This study combined laboratory tests and engineering applications, through a compressive test, tensile test, flexural test, and impervious test, for six gradation cases of HD and HP ARGFs, and studied the mechanical properties of concrete under different fiber gradation cases. In addition, the effect of the gradation amount on concrete properties was analyzed. The research results were applied to the construction of the Laoshan Tunnel in Qingdao, China, and good engineering outcomes were achieved, which provide an important reference for the application of graded ARGF concrete.

## 2. Test Materials and Methods

### 2.1. Test Material and Specimen Preparation

**Figure 2:**
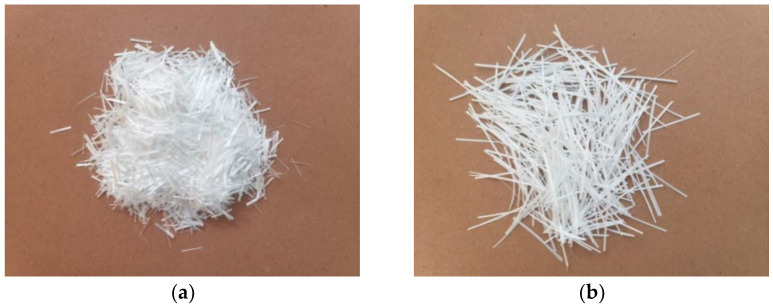
Two samples of ARGFs: (**a**) HD ARGFs; (**b**) HP ARGFs.

The cement used for the test was P.O42.5 benchmark cement produced by Yixing Tianshan Cement Co., Ltd., and two types of ARGF were used for the test, HD and HP, produced by Taishan Glass Fiber Co. (Tai’an, China). Images of the samples are shown in Figure 2. The chemical compositions of the two ARGFs were the same. The specific chemical compositions are listed in Table 1, and the basic performance parameters are listed in Table 2.

**Figure 3:**
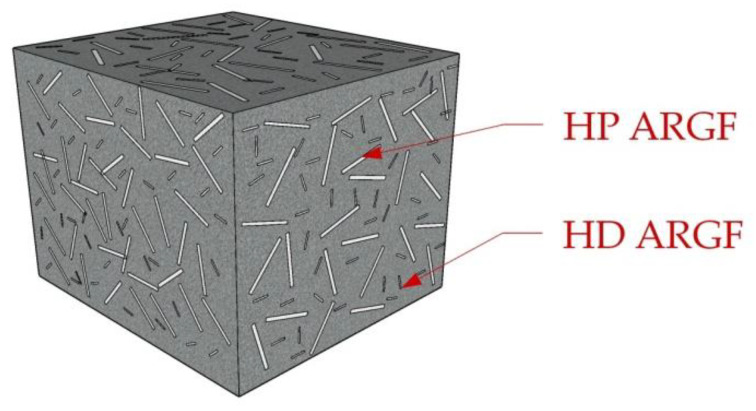
Diagram of random distribution of ARGFs.

The two types of ARGF, HD and HP, were mixed during the production of ARGF concrete test blocks and were fully mixed to ensure their even and random distribution in the concrete test blocks, as shown in Figure 3.

### 2.2. Test Scheme

#### 2.2.1. Compatibility Design

GRC is made by mixing cement, water, sand, stone, and ARGFs in certain proportions. To study the enhancement effect of HD and HP ARGFs on the mechanical properties of concrete under different grading cases, the concrete test set was classified and indicated using JZ30 for apparent concrete test blocks and FC30 for G-GRC test blocks; the concrete test block mix ratio design is shown in Table 3.

#### 2.2.2. Experimental Group Design

According to the requirements of the specification [21,22,23,24,25,26,27], to study the CS, TS, and FS of G-GRC, each experimental group contained three sample specimens, labeled using A, B, and C, respectively. However, for the IP of G-GRC, six samples were used, labeled as A, B, C, D, E, and F. The detailed test groups and sample configurations are presented in Table 4.

#### 2.2.3. Test Method

**Figure 4:**
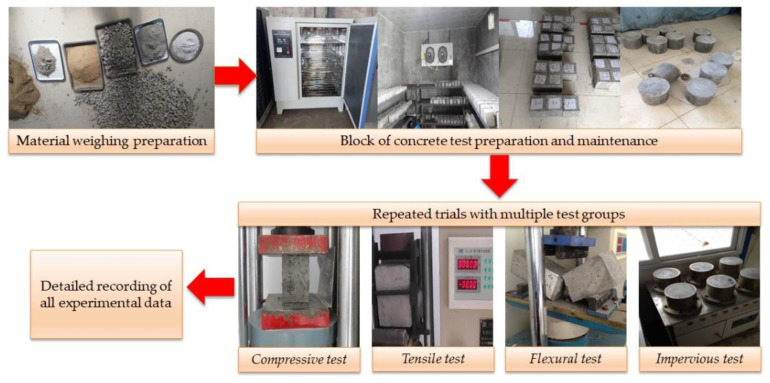
Test arrangement and procedure.

After the concrete specimens were prepared and then formed, they were left in a room with a temperature of 20 ± 5 °C and relative humidity greater than 50% for 1–2 d. After demolding, they were placed in a standard curing room with a temperature of 20 ± 2 °C and relative humidity greater than 95%. When the curing requirements are met, the test is successful. There are more international test methods for fiber reinforced concrete [28,29,30,31,32], and the Chinese CECS13:2009 standard and its auxiliary standards required for implementation [21,22,23,24,25,26,27] were selected for this test. The specific test procedure is shown in Figure 4.

## 3. Experimental Results and Analysis

Based on the above test method, the results of the compressive, splitting tensile, flexural, and impervious tests of each experimental group were plotted as visual diagrams for data analysis and analyzed as follows.

### 3.1. Analysis of CS Test Results

**Figure 5:**
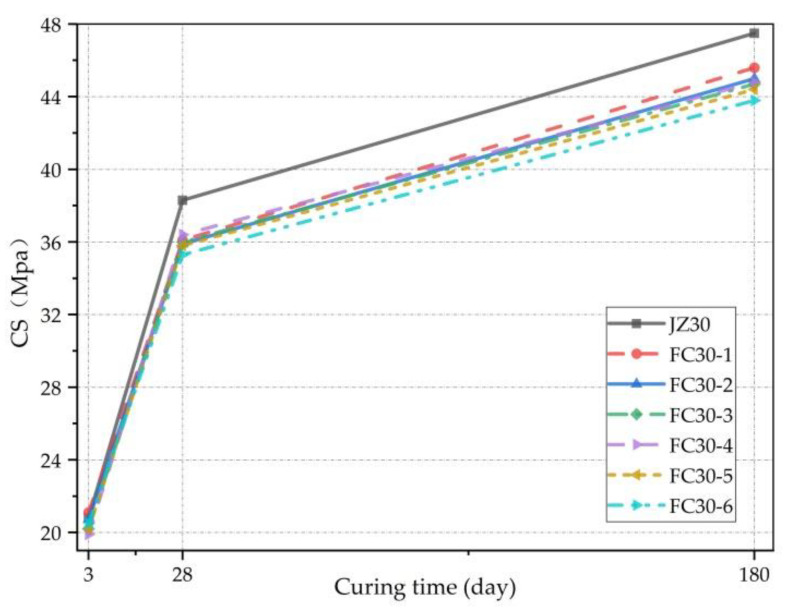
Relationship between CS and curing time.

Figure 5 clearly expresses the relationship between the CS and curing time, showing that the CSs of the G-GRC experimental groups and the ordinary concrete experimental group increased with age, as a consistent trend. The CSs increased with age then gradually eased, but compared with ordinary concrete, the CS of the G-GRC test group appeared to vary, revealing that the gradation of ARGF not only enhanced the CS of concrete but also slightly weakened against CS. These experimental results are confirmed by Wu et al. [33].

**Figure 6:**
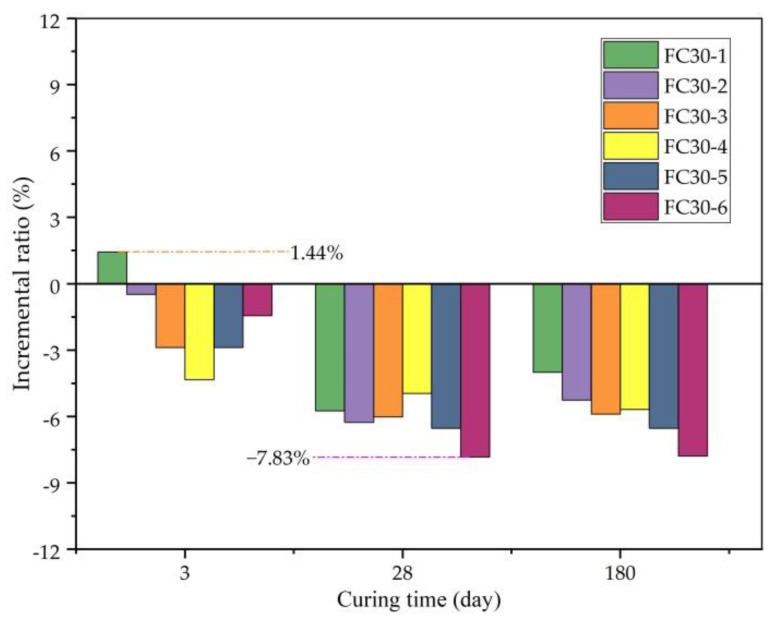
Incremental ratio of CS relative to JZ30 for each ARGF experimental group.

The CS increment ratio is the percentage of CS increment from that of normal concrete. To clearly demonstrate the degree of CS weakening, Figure 6 shows that with the increase in graded ARGF admixture, the concrete CS increment ratio fluctuates, but generally, it presents a decreasing trend. The incremental ratio of the CS of each test group was similar at the ages of 28 and 180 d, and the minimum value appeared in the FC30-6 test group at 28 d. When the incremental ratio was −7.83%, the contents of HD- and HP-type ARGFs were 0.6 and 15 kg/m^3^, respectively.

**Figure 7:**
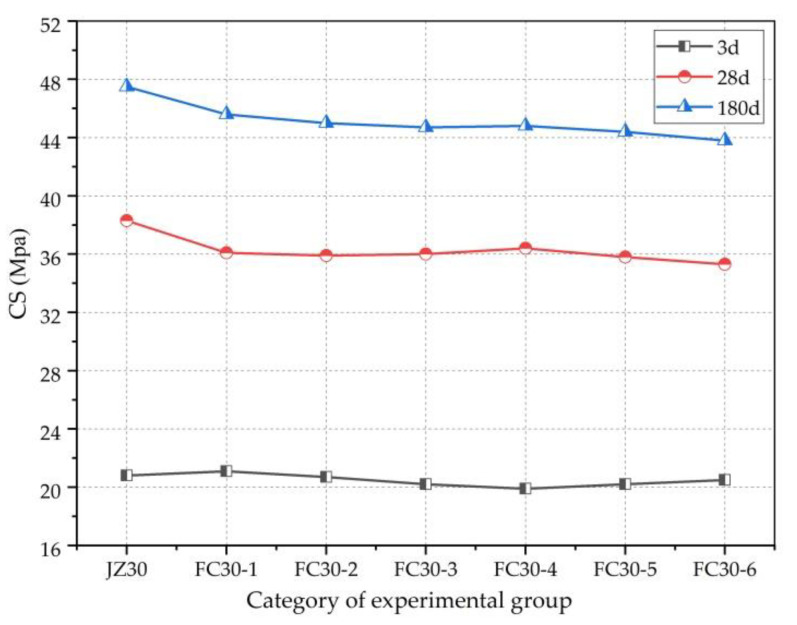
Relationship between CS and ARGF dosage.

To express the relationship between the CS and the graded ARGF admixture, Figure 7 shows that at 3 d, after admixture of the ARGFs, the change in the CS of concrete was small, and the change trend is up and down. At 28 and 180 d, after the admixture of ARGFs, the CS of concrete exhibits slight decreases. However, as the amount of ARGFs continued to increase, the CS of the concrete remained stable. This is because the length and diameter of HD ARGF are small, and the crack resistance is weak, which has less influence on the CS of concrete. It is easy to produce a weak surface at the junction of the fiber and concrete, which causes damage to the CS of concrete; therefore, compared with ordinary concrete, the CS of G-GRC generally reduces. In addition to the increase in gradation of the ARGF content, the good grip of the HP ARGF increased gradually. Nevertheless, because of the HD ARGF, its CS performance enhancement effect was limited; thus, as the dosage of ARGFs continued to increase, the CS of the concrete remained stable.

**Figure 8:**
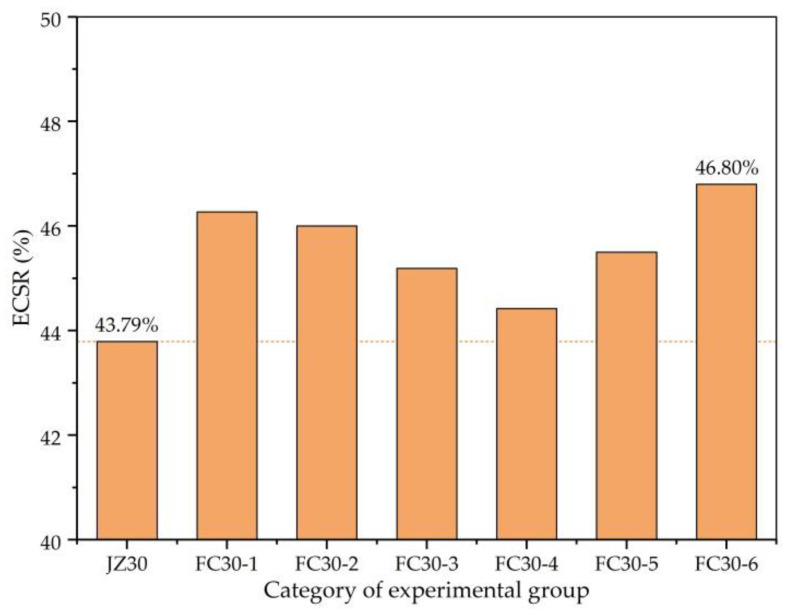
ECSR (ratio of CSs after 3 and 180 d) of each test group.

Figure 8 clearly reveals the early strength ratios (ESRs) of CS (ECSR; ratio of CSs after 3 and 180 d) for each group in the study. The larger the ESR, the earlier the concrete reaches the predetermined strength, which is of great significance in shortening the concrete construction period and other practical applications. As can be seen from the figure, the ECSR is enhanced after mixing the ARGFs and remains basically stable with the increase in graded ARGFs. The FC30-6 test group exhibited the largest ECSR with HD- and HP-type ARGF mixing amounts of 0.6 and 15 kg/m^3^, respectively, which is 46.80%, an improvement of 3.91% compared with ordinary concrete.

### 3.2. Analysis of TS Test Results

**Figure 9:**
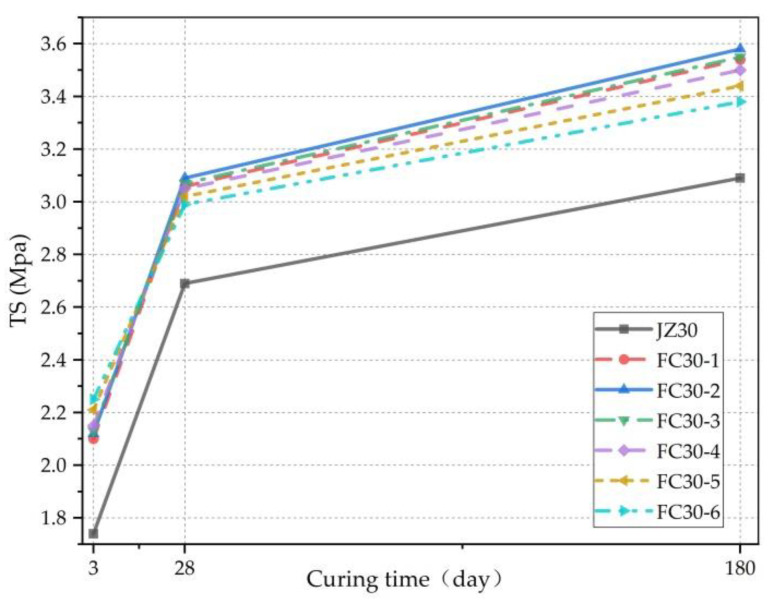
Relationship between TS and curing time.

Figure 9 clearly demonstrates the relationship between the TS and age. It can be seen that the TSs of both the G-GRC test group and the ordinary concrete test group increased with age and the changing trend was consistent; both gradually slowed with age. Meanwhile, the bridging and cracking inhibition effect of the ARGFs inhibited the generation and development of cracks in the concrete and significantly enhanced the TS of the concrete. The TS of the G-GRC test group was significantly higher than that of the ordinary concrete test group.

**Figure 10:**
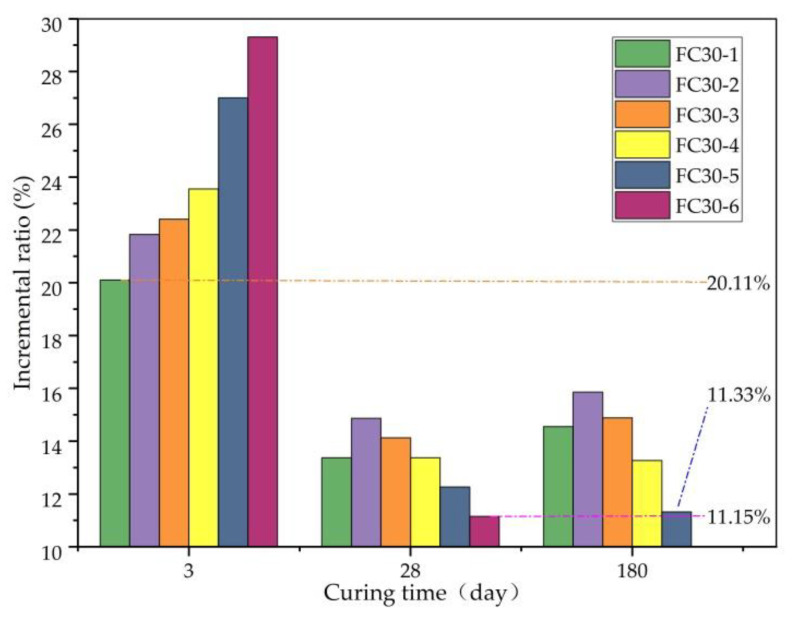
Incremental ratio of TS relative to JZ30 for each ARGF experimental group.

The TS increment ratio is the percentage of the TS increment to the TS of ordinary concrete and clearly presents the degree of TS enhancement, as shown in Figure 10. Here, it can be seen that the minimum TS increment ratios of each test group at 3, 28, and 180 d were 20.11%, 11.15%, and 11.33%, respectively, and the contribution of graded ARGF to the early TS value of concrete is the most outstanding. This is because the strength of concrete is not sufficient in the early stage, and the TS at this time mainly depends on the ARGFs. In the later stage, with the continuous improvement of concrete strength, the contribution of the ARGFs to the TS began to weaken, and the increment ratio of the TS started to decrease.

**Figure 11:**
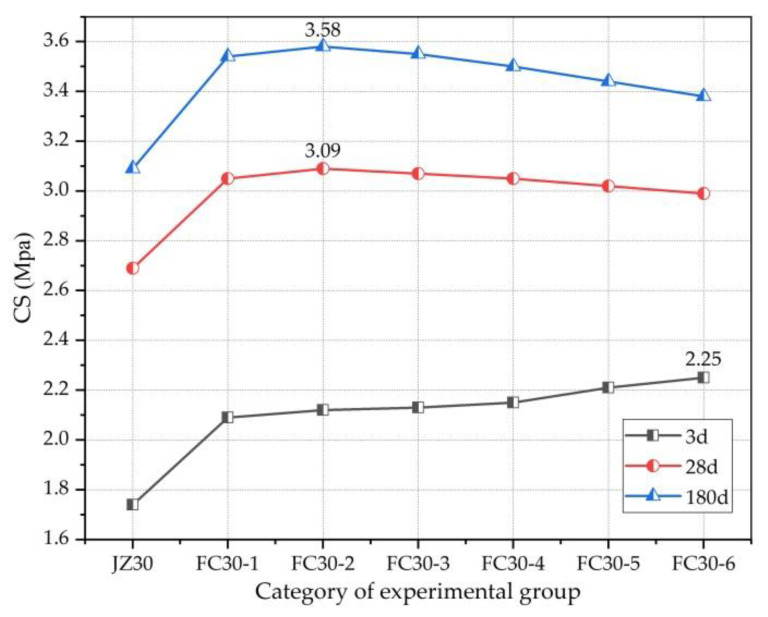
Relationship between TS and ARGF dosage.

Figure 11 clearly shows the relationship between the TS and the dosage of graded ARGF. According to the figure, at the age of 3 d, the TS increased as the dosage of graded ARGF increased. Whereas, at the ages of 28 and 180 d, the TS first increased and then decreased. This is because when the dosage of graded ARGF was too high, fiber agglomeration occurred inside the concrete test block, damaging the tensile resistance and reducing the TS. At 28 and 180 d, the maximum TS was exhibited in the FC30-2 test group. At this time, the contents of HD and HP ARGFs were 0.6 and 5 kg/m^3^, respectively, and the corresponding TSs were 3.09 and 3.58 MPa. Compared with the ordinary concrete test group, the TS was increased by 14.87% and 15.86%, respectively.

**Figure 12:**
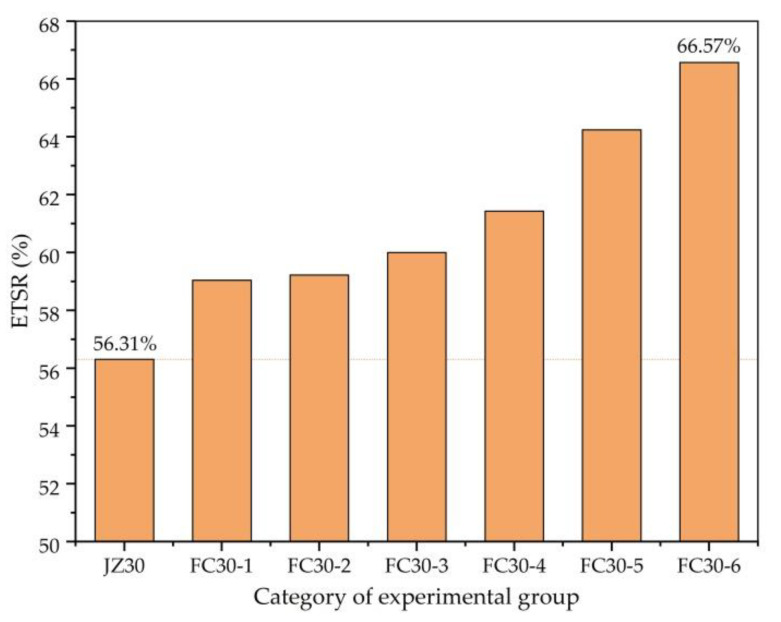
ETSR (ratio of TSs after 3 and 180 d) of each test group.

Figure 12 clearly shows the ETSRs (ratio of TSs after 3 and 180 d) of each test group. Here, it is demonstrated that graded ARGF can effectively enhance the ETSR, and the ETSR soars with an increase in the content of ARGFs. The ETSR of the FC30-6 test group with HD and HP ARGF contents of 0.6 and 15 kg/m^3^, respectively, is the largest, which is 66.57%, higher than that of ordinary concrete by 10.26%.

Concrete has the characteristics of a large CS, small TS, and high brittleness. Furthermore, in the long-term stress state, cracks easily appear without signs of crack damage. The tension–compression ratio (TCR) of concrete is the ratio of the TS to the CS of concrete, and it is an important index used to reflect concrete brittleness; the larger the TCR, the less brittle the concrete, and the stronger the toughness.

**Figure 13:**
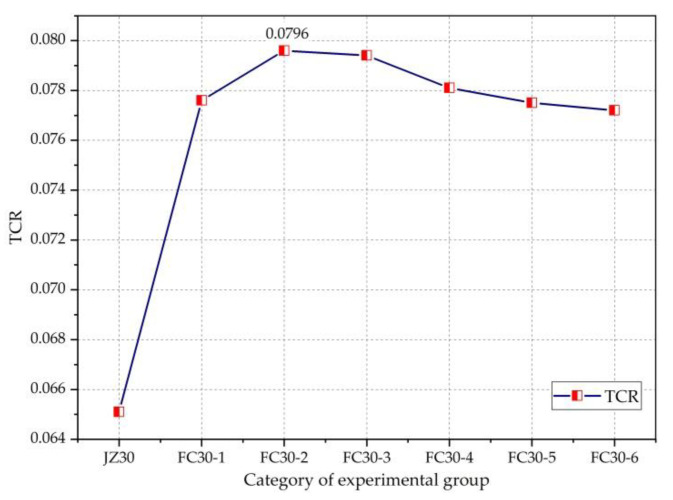
Relationship between the tension–compression ratio (TCR) and ARGF dosage.

To reveal the relationship between the TCR and the dosage of graded ARGFs, Figure 13 is drawn. It can be seen from the figure that the gradation of ARGFs clearly improved the TCR of the concrete, and with the increase in ARGF contents, the TCR first increased and then decreased. The FC30-2 test group with HD and HP ARGF contents of 0.6 and 5 kg/m^3^, respectively, exhibited the highest TCR, with a maximum value of 0.07956. Subsequently, with the continuous increase in the content of graded ARGF, the TCR began to decrease gradually, and the concrete became stronger.

**Figure 14:**
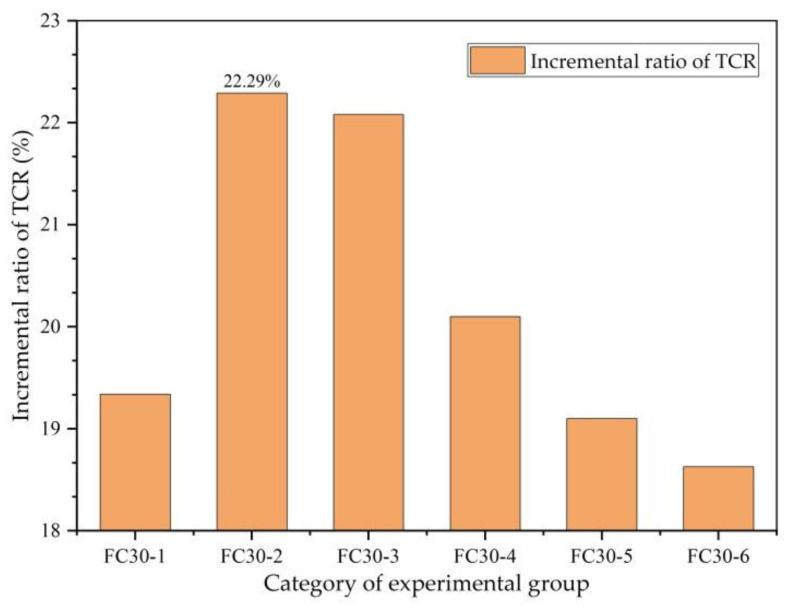
Incremental ratio of TCR to JZ30.

The increment ratio of the TCR is the percentage of the TCR increment compared with that of ordinary concrete. Figure 14 clearly shows the intensification of the TCR. It can be seen from the figure that the FC30-2 test group with the contents of HD and HP ARGFs of 0.6 and 5 kg/m^3^, respectively, exhibited the largest increment in the TCR. Compared with ordinary concrete, the TCR was increased by 22.29%, and the effect of graded ARGF on the gain of the TCR of concrete is apparent.

### 3.3. Analysis of FS Test Results

**Figure 15:**
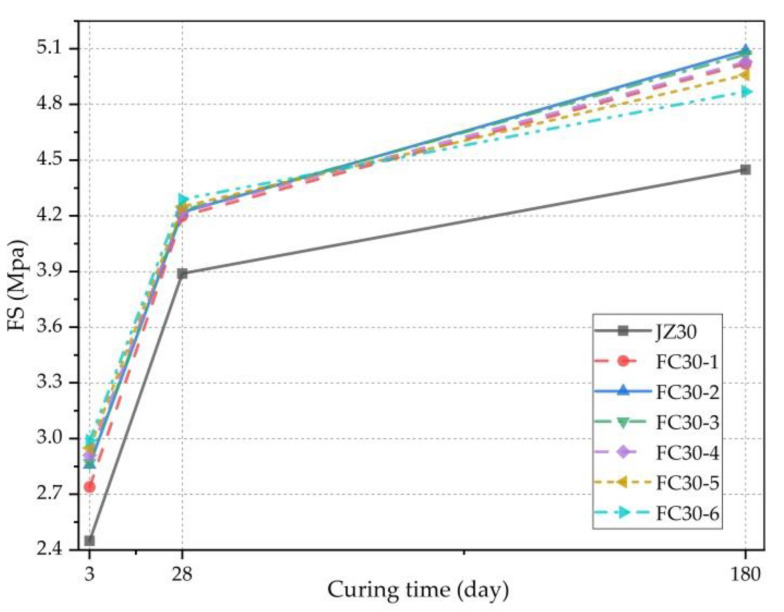
Relationship between FS and curing time.

Figure 15 clearly demonstrates the relationship between the FS and age. It can be seen here that the FSs of both the G-GRC test group and the ordinary concrete test group increased consistently with increasing age, and both gradually slowed with increasing age. Meanwhile, compared with the ordinary concrete experimental group, because the ARGFs prevented the occurrence of concrete cracks, the FS of the G-GRC test group was significantly increased and effectively enhanced the toughness of the concrete, so the flexural performance of the concrete was improved.

**Figure 16:**
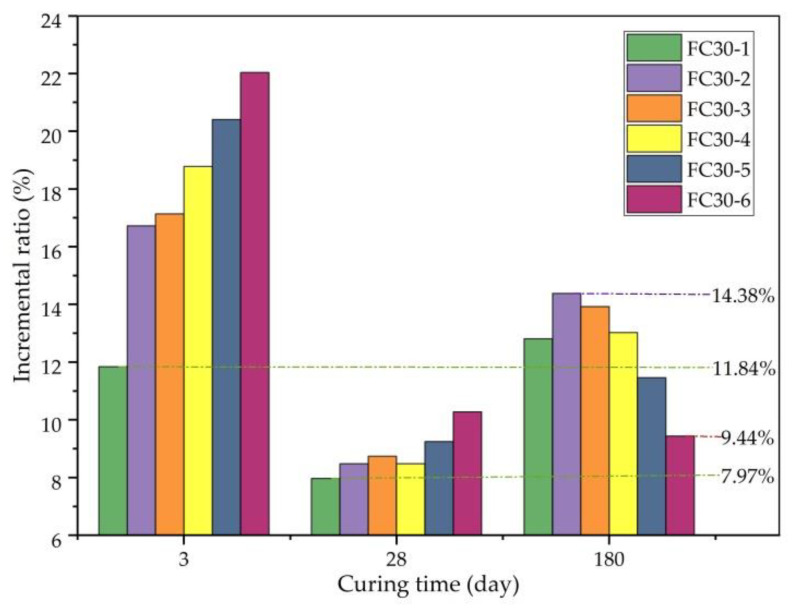
Incremental ratio of FS relative to JZ30 for each ARGF experimental group.

The increment ratio of the FS is the percentage of the increment in the FS compared with that of ordinary concrete. Figure 16 expresses the degree of enhancement of the FS. As shown, the minimum increment ratios of the FS of each test group at 3, 28, and 180 d were 11.84%, 7.97%, and 9.44%, respectively. With the increase in age, the increment ratio of the FS in most test groups revealed a changing trend from high to low, and then to high. This is because the early strength of concrete is lower. The FS mainly relies on the effect of the ARGFs; as the age increases, concrete strength increases, and the effect of the ARGFs begins to abate, but the holding force of the ARGFs remains insufficient. With a further increase in age, the strength of the concrete was further improved, the holding force of ARGF was also enhanced, and the tensile and bending performance of ARGF were better.

**Figure 17:**
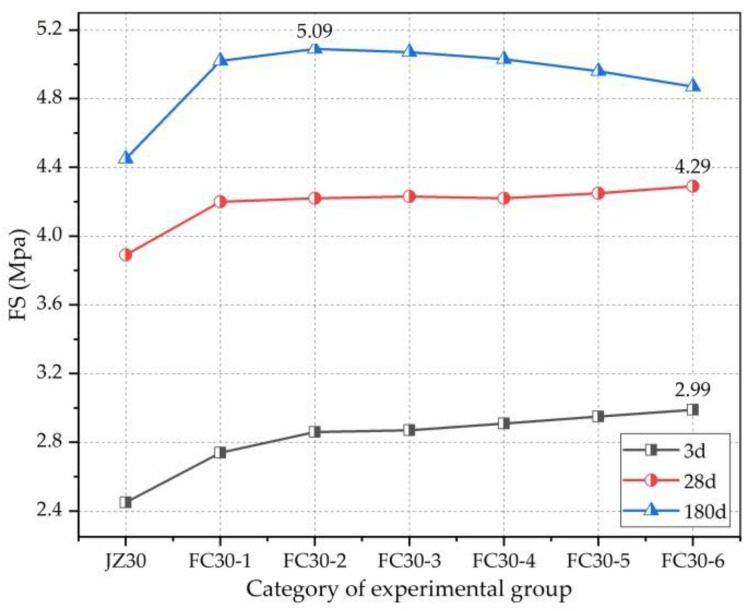
Relationship between FS and ARGF dosage.

Figure 17 clearly shows the relationship between the FS and the dosage of graded ARGFs. It can be seen that with the increase in the content of graded ARGFs, the FS of all tests exhibited a trend of steady increase at 3 and 28 d in a small range of change. However, at 180 d, the FS of each test group showed a trend of first increasing and then decreasing. The FC30-2 experimental group with HD and HP ARGF contents of 0.6 and 5 kg/m^3^, respectively, had the highest flexural strength of 5.09 MPa. The FS increased by 14.38% compared with that of the ordinary concrete test group.

**Figure 18:**
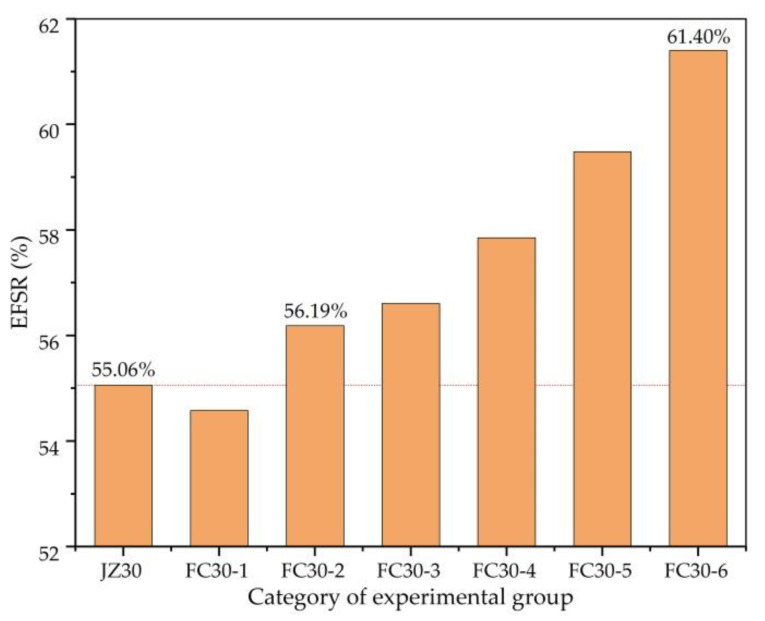
EFSR (ratio of FSs after 3 and 28 d) of each test group.

Figure 18 clearly demonstrates the ESR of the FS (EFSR) of each test group. It can be seen from the figure that when the content of graded ARGFs is low, the early strength ratio of the FS is less than that of the ordinary concrete experimental group. However, the EFSR of the concrete increases with an increase in the content of graded ARGFs. In the FC30-6 test group, the maximum EFSR value was 61.40%, which is 6.34% higher than that of ordinary concrete.

### 3.4. Analysis of IP Test Results

PH is an important index for determining the impermeability of concrete; the smaller the PH, the better the impermeability. According to GB/T50082-2009 “Test Method for Long-term Performance and Durability of Ordinary Concrete,” only the IP of concrete test blocks at 28 d was studied and analyzed.

**Figure 19:**
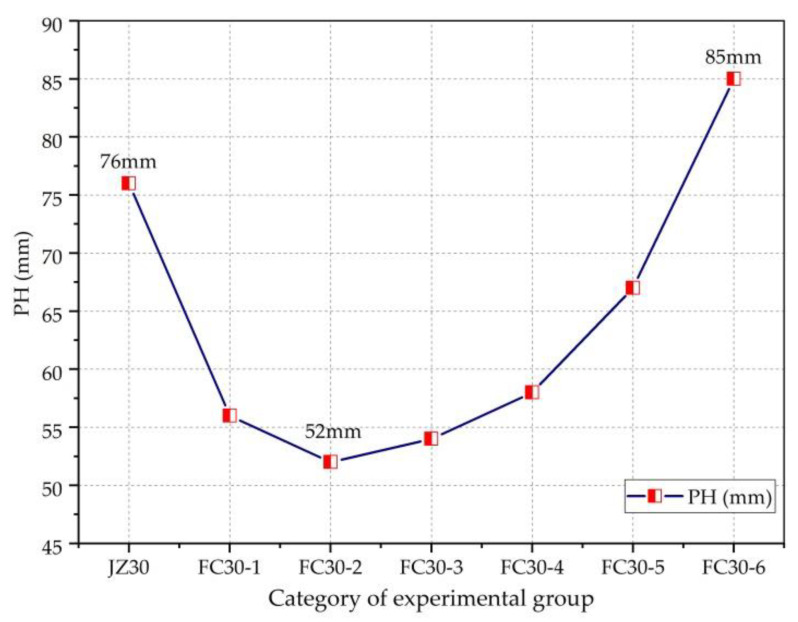
Relationship between PH and ARGF dosage.

Figure 19 clearly shows the relationship between the PH and the dosage of graded ARGFs. As can be seen from the figure, with the increase in the content of graded ARGFs, the PH first decreased and then increased. The FC30-2 test group with HD- and HP-type ARGF contents of 0.6 and 5 kg/m^3^, respectively, displayed a minimum PH of 52 mm and the best permeability resistance. This is because the ARGFs improve the denseness of the concrete as well as the pore junction, thus reducing the infiltration channels and improving the permeability of the concrete.

**Figure 20:**
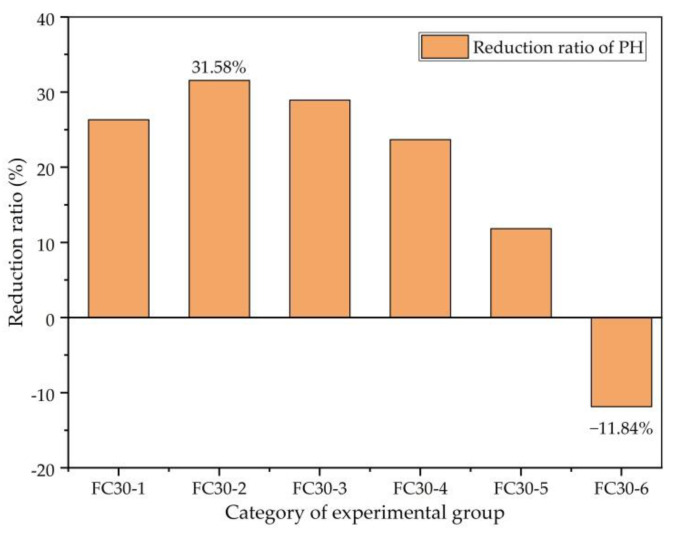
Decrease ratio of PH to JZ30.

The reduction ratio of the PH is the percentage of the reduction value of the PH compared with the PH of ordinary concrete. Figure 20 clearly shows the degree of weakening of the PH. As can be seen here, the IP reduction ratio of the FC30-2 test group with HD and HP ARGF contents of 0.6 and 5 kg/m^3^, respectively, is the largest, which was reduced by 31.58%. However, the PH reduction ratio of the FC30-6 test group with HD and HP ARGF contents of 0.6 and 15 kg/m^3^, respectively, is the smallest, which is 11.84% higher than that of ordinary concrete. This demonstrates that the appropriate amount of gradation of ARGF can significantly improve the impermeability of concrete, but in the case of excessive mixing, it will have a countereffect and damage the IP of the concrete itself.

## 4. Engineering Applications

### 4.1. Overview of Laoshan Tunnel Project

**Figure 21:**
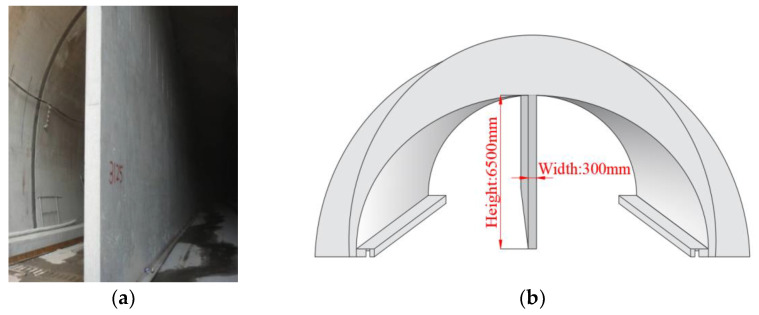
Features of the middle partition wall: (**a**) real map; (**b**) diagram of dimension parameters.

**Figure 22:**
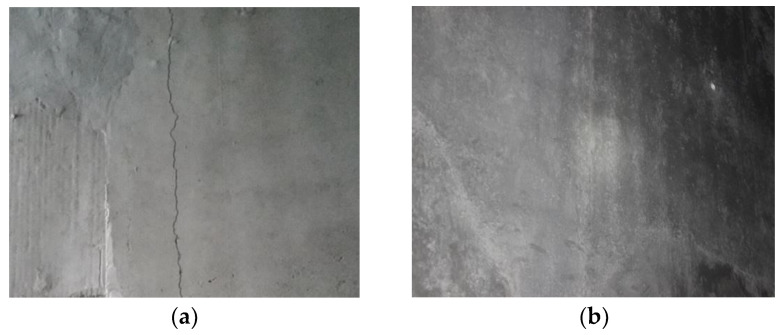
Comparison of different concrete construction effects: (**a**) ordinary concrete medium partition wall; (**b**) G-GRC partition wall.

The Laoshan tunnel is located in Qingdao City, Shandong Province, China. The tunnel is a single-hole, double-track form. The tunnel lining was designed according to the principles of the new Austrian method. It adopts a composite lining structure and was constructed by drilling and blasting. The secondary lining partition wall works through the entire tunnel. As shown in Figure 21, the middle partition wall is long, large, and thin, which easily leads to cracks in the wall, as shown in Figure 22a.

### 4.2. Application and Effect Analysis of G-GRC

According to an analysis of the laboratory experiment results, when the gradation amounts of HD- and HP-type ARGFs in concrete are 0.6 and 5 kg/m^3^, respectively, the performance is the best, and the TS and FS are improved most obviously. Therefore, this project selected HD and HP ARGF mixing contents of 0.6 and 5 kg/m^3^ for the construction site test.

After the construction of the middle partition wall with G-GRC, no large cracks appeared during the construction, and the performance of the middle partition wall improved significantly. A comparison of the partition wall with these different types of concrete is shown in Figure 22.

To further verify the strengthening effect of ARGF on the middle partition concrete, an ordinary concrete middle partition section of K35 + 325 ~ K35 + 345 miles, and a G-GRC middle partition section of K35 + 345 ~ K35 + 365 miles were selected for testing at the construction site. To ensure the accuracy of the results, the length of the middle partition wall in the each section was 20 m and the age was 180 d. Calculations were performed and the results were analyzed with reference to the technical specifications for fiber reinforced concrete structures [34], and the total crack area of the concrete partition wall (*A_cr_*) was calculated using Formula (1):

(1) $$A_{cr}=\sum_{i=1}^{n}\omega_{i}l_{i}$$

where *A_cr_* is the nominal total area of the cracks, mm^2^. The crack area of the partition wall in the fiber concrete is denoted as *A_fcr_*, and the crack area of the partition wall in ordinary concrete is denoted as *A_mcr_*.

The nominal crack width of crack *i* is recorded as *ω_i_*, i.e., the crack width near the midpoint of the crack (mm).

The length of crack *i* is given by *l_i_*, mm.

The crack reduction coefficient (*η*) can be calculated by substituting the results of Formula (1) into Formula (2):

(2) $$\eta =\frac{A_{mcr}-A_{fcr}}{A_{mcr}}.$$

The reduction coefficient calculated using Formula (2) can be evaluated according to Table 5 for the crack-limiting efficiency grade of G-GRC. In Table 6, the calculation results of cracks in the concrete partition wall are listed, including the crack area, reduction coefficient, and corresponding crack prevention efficiency grade.

It can be seen from Table 6 that the nominal total crack area of the partition wall with ordinary concrete was 1269.7 mm^2^, and the nominal total crack area of the partition wall with G-GRC was 177.2 mm^2^; the total crack area decreased by 86.04%, and the partition performance in the tunnel improved significantly. ARGF reinforced concrete has also been widely used in the secondary lining of this project, and good engineering results were obtained.

## 5. Conclusions

By mixing HD and HP, which are two types of ARGF, an experimental study on the CS, TS, FS, and IP of concrete with different fiber grades drew the following conclusions:

(1) Although the TS, FS, and IP of G-GRC were significantly enhanced, the CS decreased slightly. The grade ARGF admixture has less effect on the CS of concrete, and the decreasing value of CS remains stable, while with the augmentation of grade ARGF admixture, the TS, FS, and IP displayed trends of first increasing and then decreasing. When the contents of HD and HP ARGFs are 0.6 and 5 kg/m^3^, the bridging and cracking effects of ARGFs are the most apparent, and the overall index performance of concrete is the best.
(2) The ESRs of the CS, TS, and FS of G-GRC were all improved. For TS and FS, the ESRs increased while the TS and FS of G-GRC at all ages increased. In contrast, for CS, the ESR increased while the CS of G-GRC at all ages was reduced by different degrees. As a result, the increase in the ESRs of concrete cannot represent the improvement in the mechanical properties.
(3) According to the compression and tensile tests, owing to the substantial increase in the TS, the TCR of G-GRC was significantly improved. The TCR of G-GRC in the best-graded case is increased by 22.29% compared with that of ordinary concrete, which effectively avoids the problem of unforeseen crumbling damage of concrete due to brittleness.
(4) Experimental research on ARGF concrete was successfully applied to the construction of the Laoshan Tunnel in Qingdao, China, and achieved a good application outcome, effectively improving the quality of the project while controlling the project cost.

## 6. Discussion

This study investigated the advantages of G-GRC regarding the CS, TS, FS, and IP through indoor experiments. The proposed ARGF concrete was applied to the construction of the Laoshan Tunnel in Qingdao, China, and a good outcome was achieved. However, the action mechanism and constitutive relationship of its gradation advantages are not yet clear. Therefore, in the future, the enhanced constitutive model of G-GRC can be further studied by combining the micro-mirror method to reveal its action mechanism and to scientifically explain the test phenomenon of various properties of G-GRC.

## Figures and Tables

**Table 1:** Chemical composition of alkali-resistant glass fiber (ARGF).

Chemical Composition	SiO_2_	Na_2_O	CaO	Al_2_O_3_	ZrO_2_	TiO_2_
Content (%)	62.0	14.8	5.6	0.8	16.7	0.1

**Table 2:** Basic performance parameters of ARGF. HD: high dispersion, HP: high performance.

Species	Length (mm)	Equivalent Diameter (µm)	Fracture Strength (MPa)	Elongation at Break (%)	Modulus (GPa)	Melting Point (°C)
HD	12	14	1700	3.6	72	1580
HP	36	700	1700	3.6	72	1580

**Table 3:** Concrete test block mix ratio.

Serial Number	C30 Cement (kg/m^3^)	Sand (kg/m^3^)	Stone (kg/m^3^)	ARGF Content (kg/m^3^)		Water (kg/m^3^)	Water Reducing Agent
				**HD**	**HP**		
JZ30	370	758	1047	0	0	185	1.8%
FC30-1	370	758	1047	0.6	2.5	185	2.0%
FC30-2	370	758	1047	0.6	5	185	2.0%
FC30-3	370	758	1047	0.6	7.5	185	2.0%
FC30-4	370	758	1047	0.6	10	185	2.0%
FC30-5	370	758	1047	0.6	12.5	185	2.0%
FC30-6	370	758	1047	0.6	15	185	2.0%

**Table 4:** Experimental groups. CS: compressive strength, TS: tensile strength, FS: flexural strength, IP: impervious performance.

Project	Curing Time (d)	Experimental Group Category						
		JZ30	FC30-1	FC30-2	FC30-3	FC30-4	FC30-5	FC30-6
CS	3	A B C	A B C	A B C	A B C	A B C	A B C	A B C
	28	A B C	A B C	A B C	A B C	A B C	A B C	A B C
	180	A B C	A B C	A B C	A B C	A B C	A B C	A B C
TS	3	A B C	A B C	A B C	A B C	A B C	A B C	A B C
	28	A B C	A B C	A B C	A B C	A B C	A B C	A B C
	180	A B C	A B C	A B C	A B C	A B C	A B C	A B C
FS	3	A B C	A B C	A B C	A B C	A B C	A B C	A B C
	28	A B C	A B C	A B C	A B C	A B C	A B C	A B C
	180	A B C	A B C	A B C	A B C	A B C	A B C	A B C
IP	28	A B C	A B C	A B C	A B C	A B C	A B C	A B C
		D E F	D E F	D E F	D E F	D E F	D E F	D E F

**Table 5:** Evaluation standard of crack limiting efficiency grade.

Crack Limiting Performance Level	Evaluation Criteria
Level 1	η≥0.70
Level 2	0.55≤η≤0.70
Level 3	0.40≤η≤0.55

**Table 6:** Observation results and analysis of cracks in the middle wall.

Monitoring Range	*A_mcr_*/*A_fcr_*	*η*	Crack Limiting Performance Level
Ordinary concrete middle partition section (K35 + 325 ~ K35 + 345)	1269.7	0.86	Level 1
G-GRC middle partition section (K35 + 345 ~ K35 + 365)	177.2		

## Data Availability

The data presented in this study are available on request from the corresponding author.

## Nomenclature

**ARGF**	Alkali-resistant glass fiber
**HD-ARGF**	High-dispersion alkali-resistant glass fiber, an engineered alkali-resistant short-cut fiber that can be well-dispersed in fresh concrete
**HP-ARGF**	High-performance alkali-resistant glass fiber, a type of engineered alkali-resistant short-cut fibers composed of single filaments glued together to form a strand
**G-GRC**	Graded glass fiber reinforced concrete, comprising different types of ARGFs mixed into the concrete in a certain ratio
**CS**	Compressive strength
**TS**	Tensile strength
**FS**	Flexural strength
**IP**	Impervious performance
**PH**	Penetration height
**TCR**	Tension–compression ratio
**ECSR**	Ratio of CSs after 3 and 180 d for each experimental group; the early strength ratio of CS
**ETSR**	Ratio of TSs after 3 and 180 d for each experimental group; the early strength ratio of TS
**EFSR**	Ratio of FSs after 3 and 28 d for each experimental group; the early strength ratio of FS
$A_{cr}$	The nominal total area of cracks
$A_{mcr}$	The nominal total crack area of a partition wall in ordinary concrete
$A_{fcr}$	Nominal total crack area of partition wall in graded glass fiber reinforced concrete
η	Reduction factor of concrete crack
